# Reports of long-lasting insecticidal bed nets catching on fire: a threat to bed net users and to successful malaria control?

**DOI:** 10.1186/1475-2875-13-247

**Published:** 2014-06-28

**Authors:** Marc Egrot, Roch Houngnihin, Carine Baxerres, Georgia Damien, Armel Djènontin, Fabrice Chandre, Cédric Pennetier, Vincent Corbel, Franck Remoué

**Affiliations:** 1 MIVEGEC, Maladies Infectieuses à Vecteurs, Ecologie, Génétique, Evolution et Contrôle, IRD 224-CNRS 5290-Universités de Montpellier 1 & 2, Montpellier, France; 2 Centre de Recherche Entomologique de Cotonou (CREC), Cotonou, Bénin; 3 Département de Sociologie et d’Anthropologie, Université d’Abomey-Calavi, Abomey-Calavi, Bénin; 4 MERIT-UMR 216: Mère et enfant face aux infections tropicales, IRD, Paris, France; 5 Centre d’Étude et de Recherche sur le Paludisme Associé à la Grossesse et l’Enfance (CERPAGE), Cotonou, Bénin; 6 Faculté des Sciences et Technologies, Université d’Abomey-Calavi, Abomey-Calavi, Bénin; 7 Department of Entomology, Faculty of Agriculture at Kamphaeng Saen, Kamphaeng Saen Campus, Kasetsart University, Thailand

**Keywords:** Malaria, Bed nets, Anthropology, Fires, Use rate, Communication strategy

## Abstract

**Background:**

One of the control tools to reduce malaria transmission is the use of LLINs. However, several studies show that household bed net use is quite low. A study was developed to better understand the cultural factors that might explain these gaps in Benin. One reason mentioned is that bed nets can catch on fire and cause harm. This paper presents a summary of these findings, their analysis and the ensuing issues.

**Methods:**

This anthropological study is based on an inductive qualitative approach, including 91 semi-structured interviews conducted from July 2011 to March 2012 in a health district in Southern Benin.

**Results:**

Fifty-six persons stated that bed nets can catch on fire but do not always refer to specific facts. However, 34 of the 56 people narrate specific events they heard or experienced. 39 accounts were geographically located and situated in time, with various details. In 27 situations, people were burned, for which 12 people reportedly died.

**Discussion:**

The disparity between these results and the dearth of bibliographic documentation in the initial search prompted a more in-depth literature review: 16 contributions between 1994 and 2013 were found. Bed net fires were noted in 10 countries, but it is impossible to ascertain the frequency of such events. Moreover, bodily harm can be significant, and several cases of death attributed to bed net fires were noted.

**Conclusions:**

Indisputably, the use of bed nets to reduce the impact of this terrible disease is an optimal control method. However, the perception that LLINs have a potentially negative effect hinders the use rate in the real world, at least for some. If some people fear the risk of fires, this possibility must be addressed during information and prevention sessions on malaria, with a communication strategy tailored to specific social contexts. Moreover, all possible measures should be taken to limit the harm suffered by individuals and their families.

## Background

One of the major vector control tools to reduce malaria transmission is the large-scale use of insecticide-treated bed nets (ITNs). Their efficacy to reduce mosquito-to-human contact, Anopheles vector densities or malaria transmission has been long documented since its discovery in the eighties [1,2]. The first study of the efficacy of manufactured long-lasting insecticide-treated bed nets (LLINs) was a phase III trial conducted in Côte d’Ivoire [3-5]. This efficacy was later confirmed to reduce malaria incidence in areas where malaria vectors were either susceptible or resistant to insecticides [6]. The World Health Organization (WHO) has recommended using LLINs for about ten years as one of the three priorities that malaria endemic countries must address [7].

However, several qualitative studies conducted during the last decade demonstrate that household bed net use is quite low, even in the period following implementation of this preventive medical object. Between 2003 and 2006, national surveys were conducted in 15 sub-Saharan African countries specifically on ITN coverage and use estimates for children under five years of age and pregnant women [8]. These studies found that, the night before the survey, the bed net use rates ranged from 1.5% to 20% for children under five years despite a coverage rate (bed nets/household) between 3.3% and 42.0%; and 1.1% to 19.7% for pregnant women despite a coverage rate between 3.3% and 42.2% [8].

In 2008–2009, a project in the Ouidah-Kpomassè-Tori Bossito (OKT) health district in southern Benin indicated an average ITN coverage (bed nets/sleeping space) of 75% with a 46% use rate the night before the survey for the general population and 54% for children under five years [9,10]. In 2011, the EVALUT project shows that 80% of households in this same OKT health district have LLINs for children under five years, but only 29% of them slept under one the entire preceding two weeks before the survey and 37% the night before [11]. These studies showed that mass distributions of bed nets did not imply their widespread nor correct use as recommended. The prevention policy’s efficacy clearly depends on how individuals perceive and use the object.

For this reason, an anthropological approach was developed to better understand the cultural factors that might explain these gaps between LLIN use and coverage rates in Benin. Among other topics, a programme entitled “Anthropology of Malaria Control Methods” (see Acknowledgments) examines current trends in how decision-making processes regarding the use of LLINs are socially constructed. This paper focuses on one striking result: one reason mentioned by some people to explain a possible refusal to use bed nets is that LLINs can catch on fire and cause serious material damage and bodily harm, or even death. This article presents the following summary of these results, their analysis and the ensuing issues that arise from them.

## Methods

Using methods based in ethnology with an inductive qualitative approach, semi-structured interviews were conducted using an interview guide in OKT health district. From July 2011 to March 2012, 91 interviews were conducted in French and local languages. These interviews were translated when needed and then transcribed into word processing documents. The data were then sorted by theme before undergoing analysis.

The survey guide that was originally implemented did not include a specific item on the potential risk of nets burning. In fact, nothing in the team’s initial literature review indicated a need to include this issue. Nevertheless, during the interviews, six persons spontaneously mentioned that bed nets could catch on fire and cause material damage or bodily harm in response to a general question on the drawbacks related to their use. Therefore, the anthropological inductive approach required that a specific question be added to the interview guide, beyond the general issue of disadvantages to using bed nets.

## Results

**Figure 1:**
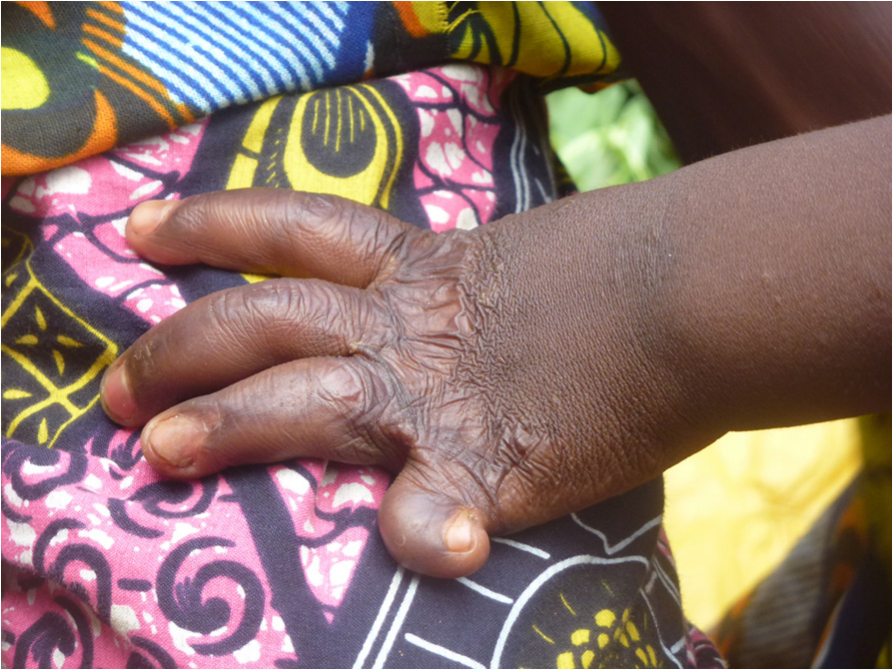
Sequelae of burns: Afissa’s left hand, side view (M Egrot, IRD, Nov 2011, Benin).

**Figure 2:**
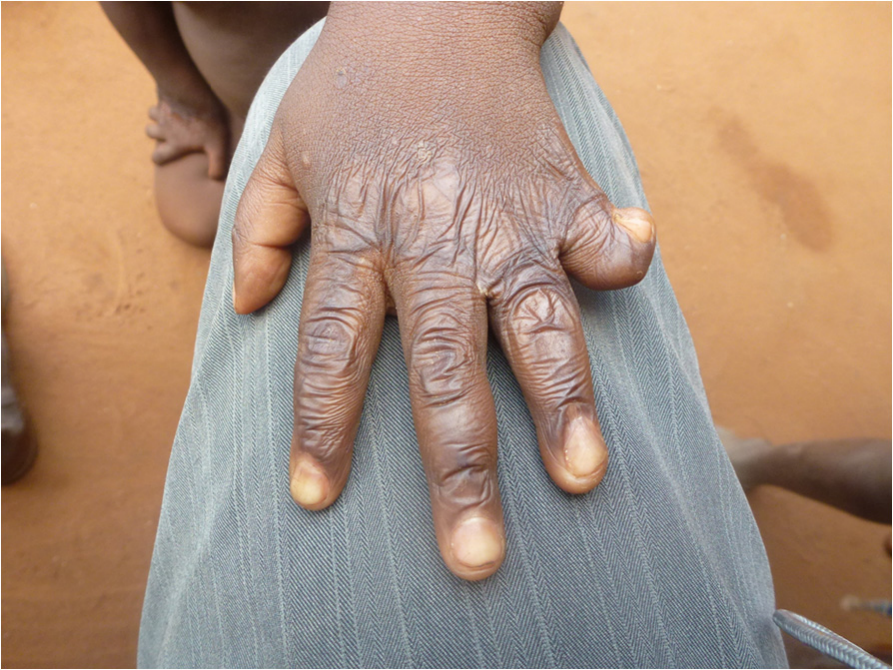
Sequelae of burns: Afissa’s left hand, dorsal view (M Egrot, IRD, Nov 2011, Benin).

**Figure 3:**
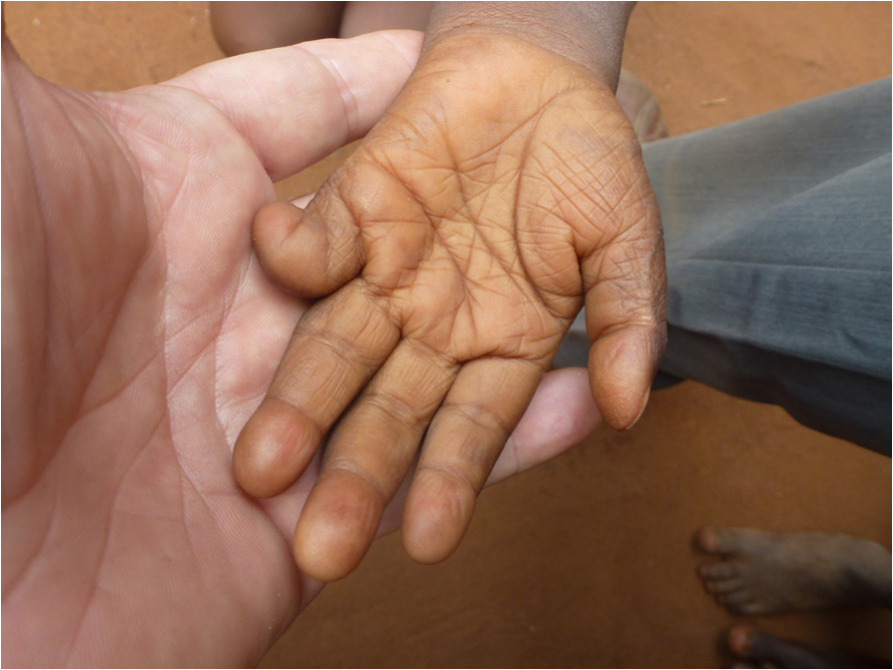
Sequelae of burns: Afissa’s left hand, palm view (M Egrot, IRD, Nov 2011, Benin).

**Figure 4:**
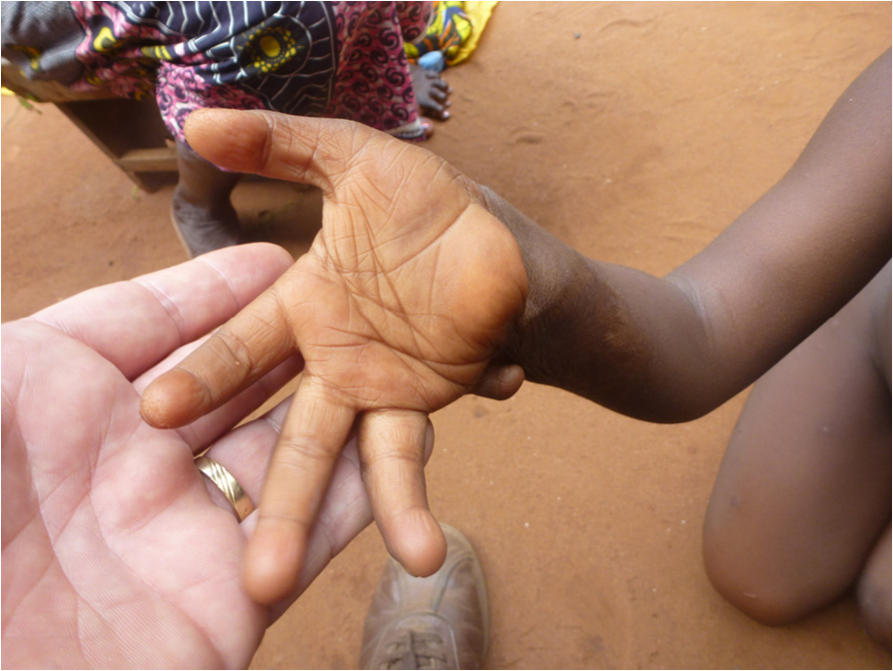
Sequelae of burns: amputation of the fifth finger of Afissa’s right hand, palm view (M Egrot, IRD, Nov 2011, Benin).

**Figure 5:**
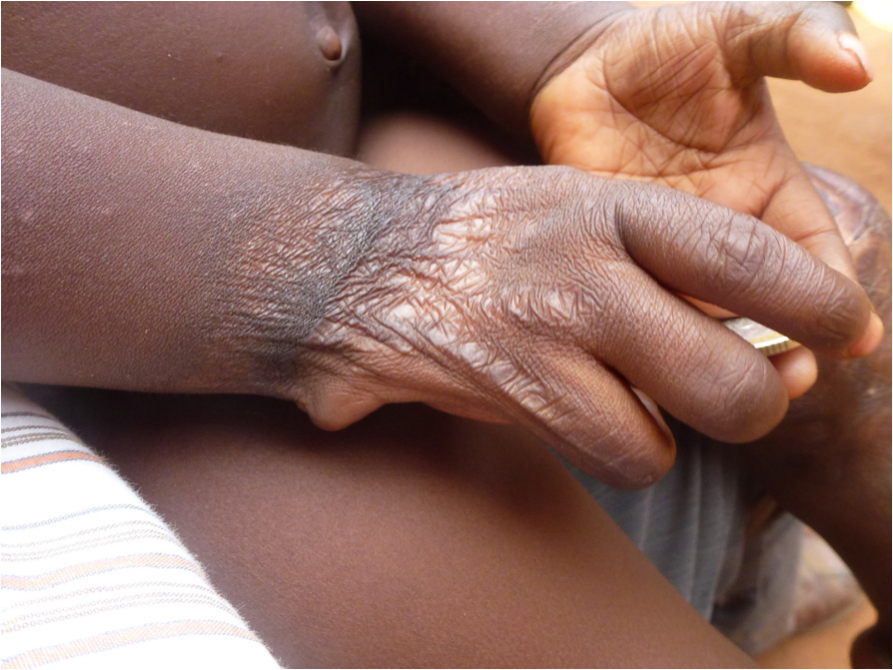
Sequelae of burns: amputation of the fifth finger of Afissa’s right hand, dorsal view (M Egrot, IRD, Nov 2011, Benin).

**Figure 6:**
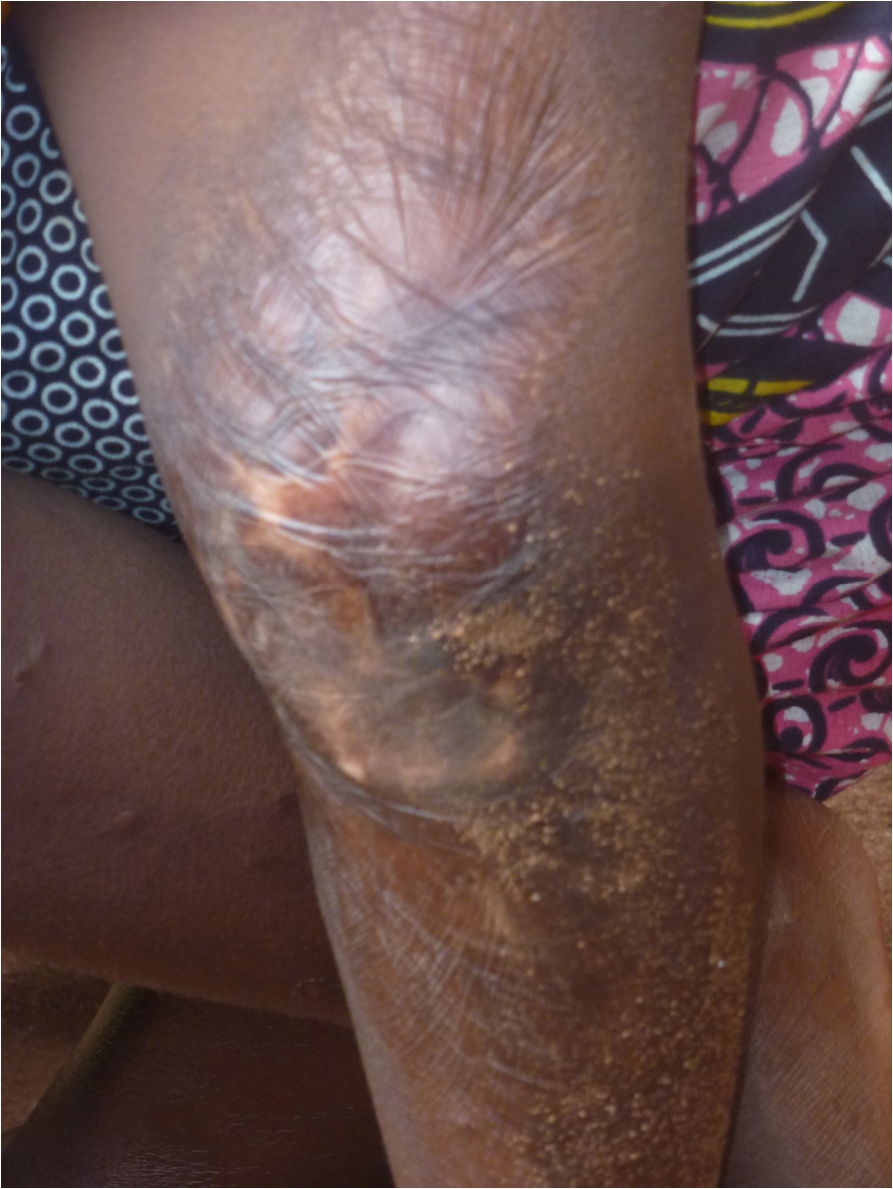
Sequelae of burns: Afissa’s left knee (M Egrot, IRD, Nov 2011, Benin).

**Figure 7:**
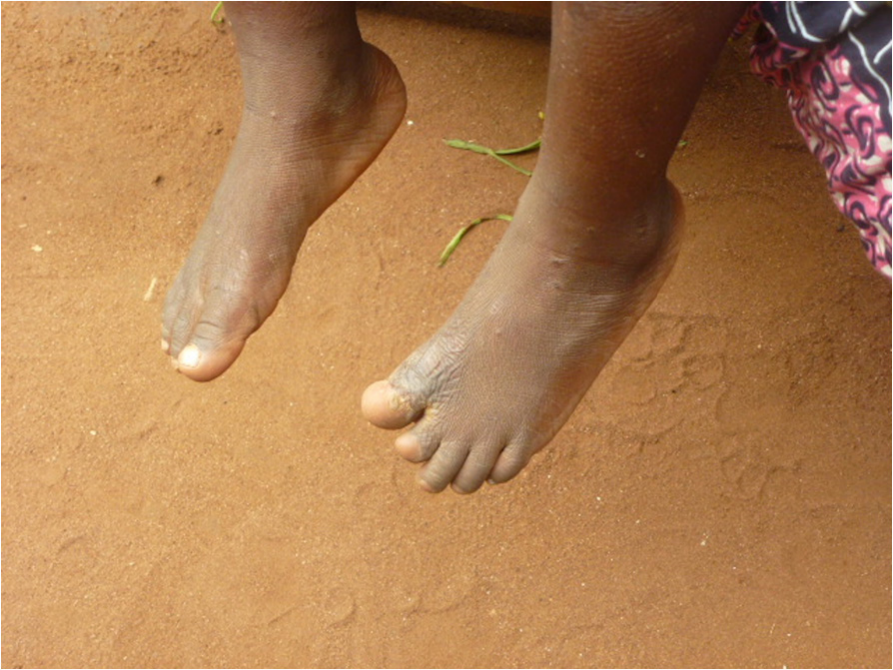
Sequelae of burns: amputation of the distal phalanges of the fourth and fifth toes (M Egrot, IRD, Nov 2011, Benin).

Of the 91 interviewees, 35 did not mention bed nets catching on fire or responded to a specific question, stating they had never heard about bed nets catching on fire. Of the remaining 56 persons, 22 stated that bed nets can catch on fire but limited their comments to generalities without referring to specific facts. By contrast, 34 people illustrated their point by referring to events they heard about or experienced, with some providing several supporting accounts (up to four in the same interview). Eleven of these accounts involved a vague memory of a fact heard in their communities or on the radio. However, 39 accounts reported by 30 persons were geographically located and situated in time, with details describing the circumstances surrounding the onset of the accident and/or its consequences. These accounts indicated 27 situations where people were burned by their bed nets, for which 12 people died. Eight of the interviewees personally knew a victim of fires attributed to bed nets catching fire. To date, the research team was able to meet with two of these individuals: a woman who suffered burns on her torso, shoulder and arm and a four-year-old girl who suffered burns at the age of one month (*cf*. The story of Afissa below and Figures 1, 2, 3, 4, 5, 6 and 7). In these narratives, the cause of the fire was nearly always related to indoor use of a lantern or candle that accidentally came into contact with the bed net. Another narrative reported a fire caused by a short-circuit on an extension cord with exposed wires. Several accounts recall how quickly the fire started and spread, along with the distinctive combustion (“it melts”, “it sticks to the skin” and “it’s like gasoline”) and the extent of material damage (burned houses, furniture or motorbikes) or bodily harm, including the 12 deaths.

### The story of Afissa

In an interview (Oct. 2, 2011), a mother recounts an incident that occurred two years earlier in Cotonou involving her daughter Afissa (Fictitious name), then aged one month. While the mother was in the shower, one of Afissa’s brothers was playing and tipped over a lantern. The mosquito net installed above the bed where Afissa slept caught on fire, burning the strings that attached it to the walls and the clothes hanging on them. Hearing the older brother’s cries, the neighbors intervened to extinguish the flames and remove the burning objects (mattresses, sheets, etc.). Nobody knew that a child was enclosed in the charred cloth. At the mother’s request, the straitjacket enclosing the child was torn. It was several minutes before anyone realized that the child was alive when they heard a sudden cry. According to the mother, Afissa’s entire body and hair was burnt. She remained in a health center for a few days, but lack of money kept her stay short. The mother applied ointments for months on her daughter’s burns. Growing up, the scarring has diminished, and her hair has almost completely grown back. Today, bodily sequelae are visible on the hands, feet, left knee and abdomen, including amputation, deformation and atrophy (see photo below).

The disparity between these results and the dearth of bibliographic documentation in the initial search prompted a more in-depth literature review.

## Discussion

A second bibliographic search focused specifically on nets burning, using the PubMed, ScienceDirect and Web of Knowledge databases. In total, 16 contributions published between 1994 and 2013 were found: five were related to social sciences. They only mentioned burning bed nets as a reason given to explain the risks related to their use in Benin [12], Colombia [13], Burkina Faso [14], Tanzania [15] and Papua New Guinea [16].

A 2002 evaluation of bed net implementation in Uganda among displaced persons showed that after two years, 2.5% of the bed nets had burned [17]. In a double-blind controlled trial in Thailand, 1.2% bed nets (4/350) burned in 1991 [18]. In a study in Kenya, the authors note that damaged bed nets were considered “worn”, notably “by fire” [19] without further details. A more recent study in Kenya inferred that 50% of net damage between 2007 and 2010 is due to burn holes and that some bed nets in the early field trials were missing because they burned. This article also reports that after one month, among the 35 lost LLINs (out of 398), three bed nets had been discarded because of fire damage [20]. One medical article written by two surgeons in Hong Kong reported two cases of children burnt on 65% and 75% of their bodies in 2005. The authors found it “surprising that no reports exist for mosquito-net related burns in the indexed medical literature”. They cited three references in the grey literature [21]: a film targeting prevention with “common burn scenarios”, including “mosquito-nets burns” with the message “open flame and mosquito nets don’t mix. Learn not to burn!” [22]; a report including an account of a “bed net fire having caused the death of a child” in 2005 in Sri Lanka [23]; and a WHO document citing fire hazard as a “negative idea” that could “hamper the introduction of LNs”. Further on, a passage pointed out: “polyester nets are more dangerous if they catch fire, because they melt causing severe burns on the skin” [24].

A WHO-RBM publication following an experts meeting on “Specifications for netting materials” addresses the issue of flammability in these terms as early as 2001. A chapter specifically entitled “Fire safety” explains: “Several specifications and test methods exist. Flammability is not the sole concern since toxic gases are also produced when burning […]. No fire retardant is normally added to polyester. Polyester is Class 1 in the American test; to raise the specification to Class 3 would require the use of retardant and this would double the price of the net. No member of the committee had heard so far of an accident involving burning polyester nets. It was recommended that an investigation into whether fire safety properties are stable over time should be undertaken” [25].

This report is particularly interesting because it seems that to date, none of the participants in this informal consultation (manufacturers, technical institutes, UN agencies, researchers) seems to have heard of bed net fires. Moreover, the takeaway assertion of this report - that the use of a fire retardant “would double the price of the net” - would be greatly exaggerated, at least today, since low-cost flame retardant barriers seem to exist [26].

Ten years after the publication of this report, two guidelines for testing or monitoring the durability of LLINs [27,28] recommend determining whether an open flame is used in the home for cooking or lighting and counting the burn holes observed on bed nets. (Burn holes and bed net fires are never mentioned in the previous 2005 version). The fact that the nets can burn therefore seems to gradually become a growing concern. However, these recommendations only propose assessing the bed net damage, not the harm suffered by users. Lastly, these guidelines do not propose calculating the percentage of bed nets that are missing because they have totally burned (as was done in the recent research in Kenya [20]). However, it is these incidents that are most likely to cause bodily harm or death.

WHO estimated that the global incidence of malaria dropped by 17% in the decade between 2000 and 2010. Its mortality rate has dropped 25% during the same period and 33% in Africa. The use of “mosquito-nets to reduce the lethal impact of this terrible disease”, which is too often deadly, cannot be underestimated, as the two surgeons from Hong Kong have so aptly highlighted [21].

Nevertheless, the current popularity of this preventive medical object and its usefulness in public health should not cause any unnecessary reluctance to take into account discourses about potential bed net fires. Given the many testimonies on this topic in this study (56/91 respondents), the team sought to determine at the beginning of the programme whether the discourses were based on rumors or real events. However, the results required that the role that rumors could actually play in the social construction of rejecting LLIN use be put into perspective, or even be dismissed. Yet, the growing frequency of stories, the precision of some accounts and discussions with eyewitnesses or victims gradually indicated that some of the fears expressed were based on actual facts. The bibliographic references that were found, despite being scant and vague, indicate that this issue arises in other contexts.

Another issue is the causal link established between the presence of a bed net and a fire. In popular representations, this does not necessarily mean that the occurrence of the second was in fact caused by the first. However, several accounts are specific about circumstances surrounding the fire, and various interviews attest to how quickly bed nets catch fire. Moreover, among all the hypotheses, at least one stands out *a priori*. In recognizing the narratives of social actors, the presence of an LLIN in the house could certainly be an exacerbating factor in fire risk, especially in homes using open flames for lighting.

The mention of bed net fires in the interviews or literature raises a series of issues in the field of public health, independent of the real or supposed issue of bed net flammability. The assumption that bed nets can burn arose in the discourses as one of the drawbacks of using bed nets. This fear does not necessarily appear to lead to reluctance or refusal to use them. On the contrary, many people who mention this risk or who experienced it as witnesses or victims declare they always use the object. Nevertheless, this perception of a potentially negative effect of ITNs may hinder the use rate in the real world. And if some people fear the risk of fires, this possibility must be addressed during information and prevention campaigns on malaria, with a communication strategy tailored to the relevant social contexts. Yet the health workers responsible for communication on malaria and bed nets are not currently equipped to address people’s concerns on this specific point. Hence, it would be desirable that they could receive advance training founded on confirmed and documented knowledge.

Lastly, several questions about the actual flammability of bed nets must also be asked. Even though literature on the subject is insufficient, and publications that refer to the issue generally lack precision, three points stand out:

– Such events were noted in several countries (Benin, Burkina Faso, Cambodia, Colombia, Hong Kong, Kenya, Uganda, Papa New Guinea, Sri Lanka and Thailand).

– It is impossible to ascertain the frequency of such events based on current knowledge. Only the evaluation in Uganda [17] and the trial in Thailand [18] estimated frequency: 2.5% and 1.2%, respectively. But the Thai study is old and dealt with cotton bed nets, while the Spencer study investigates Permanet^®^ bed nets but used in a specific context: a refugee camp.

– Bodily harm can be significant, and several cases of death attributed to bed net fires were noted. Therefore, it is important to do everything possible to limit the harm suffered by individuals and their families.

## Conclusions

Massive and widespread LLIN distribution in recent years meant that the number of bed nets delivered in Africa rose from 5.6 million in 2004 to 145 million in 2010. Between 2008 and 2010, 294 million bed nets were provided, with an additional 100 million in 2011 [29]. Whatever the actual risk of fire, this increased number of ITNs will lead to increased incidence of fire, which could hinder malaria control policies. At the same time, in order to deliver reliable and consistent information, shouldn’t the actual flammability of the currently distributed bed nets be better documented? WHO recommends assessing this, and the 2001 report already raised concerns about this issue, emphasizing “There is a need for further investigations into parameters. Flammability is important for safety, permeability for comfort” in the Introduction of Part 2 [25]. An initial step could be to propose a more systematic evaluation of the incidence of fires in trials for new bed nets or control programme evaluations. Pharmacovigilance studies could be implemented in health centers and particularly in urgent care centers or burn units. Retrospective surveys on the causes of burns could also provide useful information that is quickly accessible. Lastly, an issue raised in the WHO report probably deserves greater attention so that precise information about the comparative flammability of bed nets, variations in this flammability over time and under normal conditions of use or even on the toxic effects of fumes released during these fires is available to malaria control actors.

## Ethics approval

The EVALUT Programme was approved by the National Ethics Committee for Research in Health (Comité National d’Ethique de la Recherche en Santé; CNERS, IRB00006860) of the Benin Ministry of Health, no. 003 of 24 March 2011.

## Consent

Written informed consent was obtained from the father of Afissa for the publication of this report in Malaria Journal.

## Abbreviations

AMELPA: Anthropologie des Méthodes de Lutte contre le Paludisme/Anthropology of Malaria Control Methods; CNERS: Comité National d’Ethique de la Recherche en Santé/National Ethics Committee for Research in Health; CERPAGE: Centre d’Étude et de Recherche sur le Paludisme Associé à la Grossesse et l’Enfance/Study and Research Center on Malaria Associated with Pregnancy and Childhood; CREC: Centre de Recherche Entomologique de Cotonou/Entomoligical Research Center of Cotonou; IRD: Institut de Recherche pour le Développement/Research Institute for Development; ITNs: Insecticide-treated bed nets; LLIP: Laboratoire de Lutte Intégré contre le Paludisme/Laboratory for Integrated Malaria Control; LMI: Laboratoire Mixte International/Joint International Laboratory; LLIN: Long-lasting insecticide-treated bed nets; MERIT: Mother and child in relation to tropical infections; MIVEGEC: Maladies Infectieuses à Vecteurs, Ecologie, Génétique, Evolution et Contrôle/Infectious Diseases and Vectors, Ecology, Genetics, Evolution and Control; OKT health district: Ouidah-Kpomassè-Tori Bossito health district; RBM: Roll Back Malaria; WHO: World Health Organization.

## Competing interests

The authors declare that they have no competing interests.

## Authors’ contributions

ME wrote the manuscript. ME, RH and CB read and commented on the manuscript. GD, AD, FC, CP, VC and FR proofread the article and provided corrections. All authors read and approved the final manuscript.
